# Sperm chromatin condensation as an *in vivo* fertility biomarker in bulls: a flow cytometry approach

**DOI:** 10.1186/s40104-021-00634-7

**Published:** 2021-11-09

**Authors:** Marc Llavanera, Jordi Ribas-Maynou, Ariadna Delgado-Bermúdez, Sandra Recuero, Rodrigo Muiño, Carlos O. Hidalgo, Carolina Tamargo, Sergi Bonet, Yentel Mateo-Otero, Marc Yeste

**Affiliations:** 1 Biotechnology of Animal and Human Reproduction (TechnoSperm), Faculty of Sciences, University of Girona, C/ Maria Aurèlia Campany, 69, ES-17003 Girona, Spain; 2 Department of Biology, Unit of Cell Biology, Faculty of Sciences, University of Girona, ES-17003 Girona, Spain; 3 Department of Animal Pathology, Faculty of Veterinary Medicine, University of Santiago de Compostela, ES-15705 Lugo, Spain; 4 Department of Animal Selection and Reproduction, The Regional Agri-Food Research and Development Service of Asturias (SERIDA), E-33394 Gijón, Spain

**Keywords:** Bull, Chromatin, Chromomycin A3, Condensation, Fertility, Flow cytometry, Sperm

## Abstract

**Background:**

Genetic selection in cattle has been directed to increase milk production. This, coupled to the fact that the vast majority of bovine artificial inseminations (AI) are performed using cryopreserved sperm, have led to a reduction of fertility rates over the years. Thus, seeking sensitive and specific sperm biomarkers able to predict fertility rates is of vital importance to improve cattle reproductive efficiency. In humans, sperm chromatin condensation evaluated through chromomycin A3 (CMA3) has recently been purported to be a powerful biomarker for sperm functional status and male infertility. The objectives of the present study were: a) to set up a flow cytometry method for simultaneously evaluating chromatin condensation and sperm viability, and b) to test whether this parameter could be used as a predictor of *in vivo* fertility in bulls. The study included pools of three independent cryopreserved ejaculates per bull from 25 Holstein males. Reproductive outcomes of each sire were determined by non-return rates, which were used to classify bulls into two groups (highly fertile and subfertile).

**Results:**

Chromatin condensation status of bovine sperm was evaluated through the combination of CMA3 and Yo-Pro-1 staining and flow cytometry. Sperm quality parameters (morphology, viability, total and progressive motility) were also assessed. Pearson correlation coefficients and ROC curves were calculated to assess their capacity to predict *in vivo* fertility. Sperm morphology, viability and total motility presented an area under the ROC curve (AUC) of 0.54, 0.64 and 0.68, respectively (*P* > 0.05), and thus were not able to discriminate between fertile and subfertile individuals. Alternatively, while the percentage of progressively motile sperm showed a significant predictive value, with an AUC of 0.73 (*P* = 0.05), CMA3/Yo-Pro-1 staining even depicted superior results for the prediction of *in vivo* fertility in bulls. Specifically, the percentage of viable sperm with poor chromatin condensation showed better accuracy and precision to predict *in vivo* fertility, with an AUC of 0.78 (*P =* 0.02).

**Conclusions:**

Chromatin condensation evaluated through CMA3/Yo-Pro-1 and flow cytometry is defined here as a more powerful tool than conventional sperm parameters to predict bull *in vivo* fertility, with a potential ability to maximising the efficiency of dairy breeding industry.

**Supplementary Information:**

The online version contains supplementary material available at 10.1186/s40104-021-00634-7.

## Introduction

The dairy breeding industry selects male and females on the basis of genetic traits for increasing milk production and, more recently, their longevity and susceptibility to disease, which declines fertility rates [1, 2]. Additionally, the vast majority of artificial inseminations (AI) in cattle are conducted using cryopreserved sperm, which is known to present lower sperm quality and fertility [3]. In this regard, the prediction of bull sperm fertility using post-thawed samples is of outmost importance to maximise efficiency and profitability in the dairy breeding industry [4]. The most sensitive prediction of bull fertility rates can be achieved by inseminating a high number of known-fertility cows, but this method is very expensive, time-consuming and does not allow the analysis of a large number of animals. To overcome these drawbacks, prognosis of bull sperm fertilizing potential has traditionally been performed through conventional semen analysis (ejaculate volume, sperm concentration, morphology and motility), which is more rapid and economic. However, the use of these parameters usually present poor sensitivity and accuracy, thus showing limited value for the sector [5]. Hence, the exploration of new biomarkers with higher sensitivity and specificity in predicting *in vivo* fertility is of great interest for the dairy breeding industry [5].

In humans, evidence that sperm DNA quality is a major factor for successful fertilization and subsequent embryo development is accumulating in the literature [6–8]. Similarly, many authors suggested the association of sperm DNA integrity with sperm quality and fertility in farm animals [9–11]. While sperm DNA integrity, assessed through different methods, is the most common genetic parameter used to predict male infertility in humans [8, 12, 13], different studies showed that sperm protamine content and protamine1/protamine2 ratio may also underlie infertility [14–16]. Although chromatin might also be contributing to fertilization success in livestock, given the relevance of nucleoprotein structure integrity [17–19], it has not been well-studied as a fertility biomarker [9]. In this sense, the role of sperm chromatin condensation in production species is worth of study, since it could potentially become a new predictive tool for sperm quality and male fertility.

Sperm chromatin condensation can be indirectly evaluated by their protamine content measured through chromomycin A3 (CMA3) staining. CMA3 is a cell-permeant dye that competes with protamines to bind the DNA minor groove [20] in a Mg^2+^-dependent manner and preferentially to GC contiguous sequences [21–23]. As sperm protamine deficiency is associated to nuclear decondensation, sperm cells presenting this type of alteration are deemed to show chromatin decondensation, thus accumulating CMA3. Hence, CMA3 indirectly assess chromatin decondensation in sperm cells [24]. In humans, CMA3 staining analysed through fluorescence microscopy has been widely investigated to evaluate its predictive value for male fertility and sperm quality [16, 25]. However, although CMA3 has been studied in bulls [24, 26] and boars [27], the evaluation of this potential predictive tool in farm animals is more limited, and exclusively assessed through fluorescence microscopy. To the best of our knowledge, no studies investigating chromatin condensation through flow cytometry as an *in vivo* fertility biomarker have been conducted in bovine sperm.

As aforementioned, the vast majority of studies evaluating sperm chromatin condensation through CMA3 in mammalian species were conducted using fluorescence microscopy rather than flow cytometry. Nevertheless, it is well known that, for sperm assessment, not only does flow cytometry show a series of advantages over fluorescence microscopy, such as higher objectivity, reproducibility and sensitivity, but it also enables performing a larger number of measures in shorter time [28]. Moreover, flow cytometry allows simpler and simultaneous evaluation of many sperm parameters. In this regard, to the best of our knowledge, no study has simultaneously evaluated chromatin condensation and sperm viability through flow cytometry.

Therefore, the first aim of the present study was to evaluate chromatin condensation and sperm viability using a simultaneous double staining of CMA3 and Yo-Pro-1, thus establishing the chromatin condensation status of viable, non-viable and total sperm populations. Moreover, we also sought to explore whether sperm chromatin condensation could be used as a tool for determining *in vivo* fertility in bulls.

## Materials and methods

### Animals and ejaculates processing

A total of 25 healthy and sexually mature Holstein bulls (i.e., biological replicates; *n* = 25) from 1.5 to 2-year-old were used in the present study. Each biological replicate consisted of a pool of three independent ejaculates from the same sire, using two straws per ejaculate, and prepared prior to the assessment of sperm quality. Ejaculates were collected using an artificial vagina (internal temperature: 45 °C) at weekly intervals for 5 weeks. Animals were housed at Cenero AI centre in Gijón, Asturias (Spain), complying with all European Union regulations for animal husbandry, under standard feeding and housing conditions, to produce commercially available cryopreserved sperm straws. *In vivo* fertility was assessed through 90-day non-return rates (NRR; proportion of cows that did not return to oestrus after 90 days of AI). NRR was obtained by dividing the fertilized cows by the total number of inseminations. The average number of total inseminated cows per bull was 2,488 (with a minimum of 577 sows per bull). The distribution of bull NRRs is represented in Additional file 1: Supplementary Fig. 1. Additional approval from an ethical committee to conduct this study was not required.

Ejaculates with 2–8 mL of volume, > 10^9^ sperm per mL and > 85% of total motile sperm were subjected to cryopreservation. Sperm samples were cryopreserved using a standard procedure defined before [29]. Briefly, sperm concentration was adjusted to 92 × 10^6^ sperm per mL at 22 °C using a commercial extender (Bioxcell; IMV Technologies L’Aigle, France), and subsequently cooled at a rate of − 0.2 °C/min until reaching 4 °C. Then, sperm were equilibrated by holding at 4 °C for 3 h. Subsequently, sperm were packaged into 0.25-mL straws and cryopreserved using a controlled-rate freezer (Digit-cool; IMV Technologies), with the following cooling rates: 5 °C/min from 4 °C to − 10 °C; 40 °C/min from − 10 °C to − 100 °C; and 20 °C/min from − 100 °C to − 140 °C. Finally, straws were plunged into liquid nitrogen and stored in a nitrogen tank. Cryopreserved sperm samples were thawed at 38 °C for 20 s in a water bath and incubated at the same temperature up to 4 h. Sperm quality and functionality analyses were performed at 0 and 4 h post-thaw to evaluate both the initial quality of the samples and their resilience over time.

### Evaluation of sperm morphology

Sperm morphology was evaluated using a phase-contrast microscope at 200 × magnification coupled with a SCA® Production software (Sperm Class Analyzer Production, 2010; Microptic S.L., Barcelona, Spain). Two hundred sperm per sample were evaluated and classified as normal or abnormal sperm (abnormal head size and shape, acrosome abnormalities, folded and coiled tails, proximal and distal droplets and isolated heads).

### Evaluation of sperm motility

Sperm motility was assessed through a computer-assisted sperm analysis (CASA) system, using a negative phase-contrast field (Olympus BX41 with 10 × 0.30 PLAN objective; Olympus, Tokyo, Japan) and the ISAS software (Integrated Sperm Analysis System V1.0; Proiser SL, Valencia, Spain) set at 30 frames per second. Three μL of each sperm sample at 38 °C were loaded into a pre-warmed 20 μm-Leja chamber slide (Leja Products BV; Nieuw-Vennep, The Netherlands). A sperm cell was considered to be motile when the average path velocity (VAP) was higher than 10 μm/s, whereas a sperm cell was considered to be progressively motile if its index of straightness (STR) was higher than 70%. Two technical replicates per sample, with a total of 1,000 sperm per replicate, were analysed. Percentages of total and progressive motile sperm were used to assess sperm motility.

### Evaluation of sperm viability (SYBR-14/PI)

Sperm viability was assessed using the LIVE/DEAD sperm viability kit (Molecular Probes, Eugene, OR, USA), following the protocol of Garner and Johnson [30] with minor modifications. Briefly, 4 × 10^6^ sperm per mL were stained with SYBR-14 (final concentration: 32 nmol/L) and propidium iodide (final concentration: 7.5 μmol/L) at 38 °C in the dark for 15 min, and subsequently analysed with a CytoFLEX cytometer (Beckman Coulter; Fullerton, CA, USA). SYBR-14 fluorescence was detected by FITC channel (525/40), whereas PI fluorescence was collected through PC5.5 channel (690/50). Both fluorochromes were excited with a 488-nm laser, and no spill compensation was applied. The percentage of viable, green-stained sperm (SYBR-14^+^/PI^−^) was used to assess sperm viability.

### Evaluation of sperm chromatin condensation (CMA3/YoPro-1)

Sperm chromatin condensation was determined through double staining with CMA3, for chromatin (de) protamination, and Yo-Pro-1, for sperm viability. Stock solutions of CMA3 and Yo-Pro-1 were prepared at 500 μg/mL and 25 μmol/L, respectively. Sperm samples were diluted 1:1 (v:v) in 2 × McIlvine solution (60 mmol/L citric acid, 280 mmol/L Na_2_HPO_4_ and 20 mmol/L MgCl_2_), reaching a final concentration of 20 × 10^6^ sperm per mL. Following this, diluted samples were stained with CMA3 (final concentration: 12.5 μg/mL) at room temperature in the dark for 20 min, and subsequently diluted 1:10 (v:v) in filtered PBS. Thereafter, samples were stained with Yo-Pro-1 (final concentration: 0.2 μmol/L) for 5 min in the same conditions. Finally, diluted and stained samples were analysed with a CytoFLEX cytometer (Beckman Coulter Fullerton, CA, USA). Yo-Pro-1 was excited with a 488-nm laser and its emission was acquired with the FITC channel (525/40). On the other hand, CMA3 was excited with a 405-nm laser and its emission was acquired with the Violet610 channel (610/20). A negative control, in which CMA3 was omitted, was used to establish the CMA3^−^ population in each sample. CMA3^+^ population was determined as the sperm presenting higher fluorescence intensity than the negative control threshold. Sperm chromatin condensation was determined through percentages and mean fluorescence intensities of CMA3 in the following populations: viable, CMA3^+^ sperm (CMA3^+^/Yo-Pro-1^−^); non-viable, CMA3^+^ sperm (CMA3^+^/Yo-Pro-1^+^); and total CMA3^+^ sperm (CMA3^+^).

In order to set up the methodology, an experiment was performed using five cryopreserved bull sperm samples in which a positive control was generated through incubation with 5 mmol/L Dithiothreitol (DTT) for 45 min at 37 °C. After these incubations, samples were centrifuged at 600 × *g* for 5 min and washed in PBS three times. At this point, both samples and positive controls were subjected to the evaluation of sperm chromatin condensation described above.

### Statistical analyses

Results were evaluated and plotted using IBM SPSS Statistics 25.0 (IBM Corp., Armonk, NY, USA) and GraphPad Prism v.8 (GraphPad Software, La Jolla, CA,United States), respectively. Data were checked for normal distribution (Shapiro-Wilk test) and homogeneity of variances (Levene test) prior to statistical analysis. Subfertile (cases) and high fertility (controls) groups were established using the NRR median value. Differences between groups were tested through one-way analysis of variance (ANOVA) followed by post-hoc Sidak for pair-wise comparisons. Correlations between sperm quality parameters and chromatin condensation status were determined through Pearson coefficient. The analysis of the setting up of the CMA3 methodology through incubations with DTT was analyzed through the Wilcoxon paired samples test.

A receiver operating curve (ROC) analysis was performed to obtain the area under the curve (AUC), sensitivity, specificity and cut-off values of every sperm parameter. A principal component analysis (PCA) of sperm parameters showing good predictive value (percentage of progressively motile and viable CMA3^+^ sperm) was also performed. The resulting regression factors were used to assess its fertility predictive value through ROC analysis.

Each biological replicate was considered as a statistical case (*n* = 25). The level of significance was set at *P* ≤ 0.05, in order to consider a confidence interval of 95% (CI95%).

## Results

### Flow cytometry assessment of chromatin condensation by CMA3/Yo-Pro-1

**Fig. 1:**
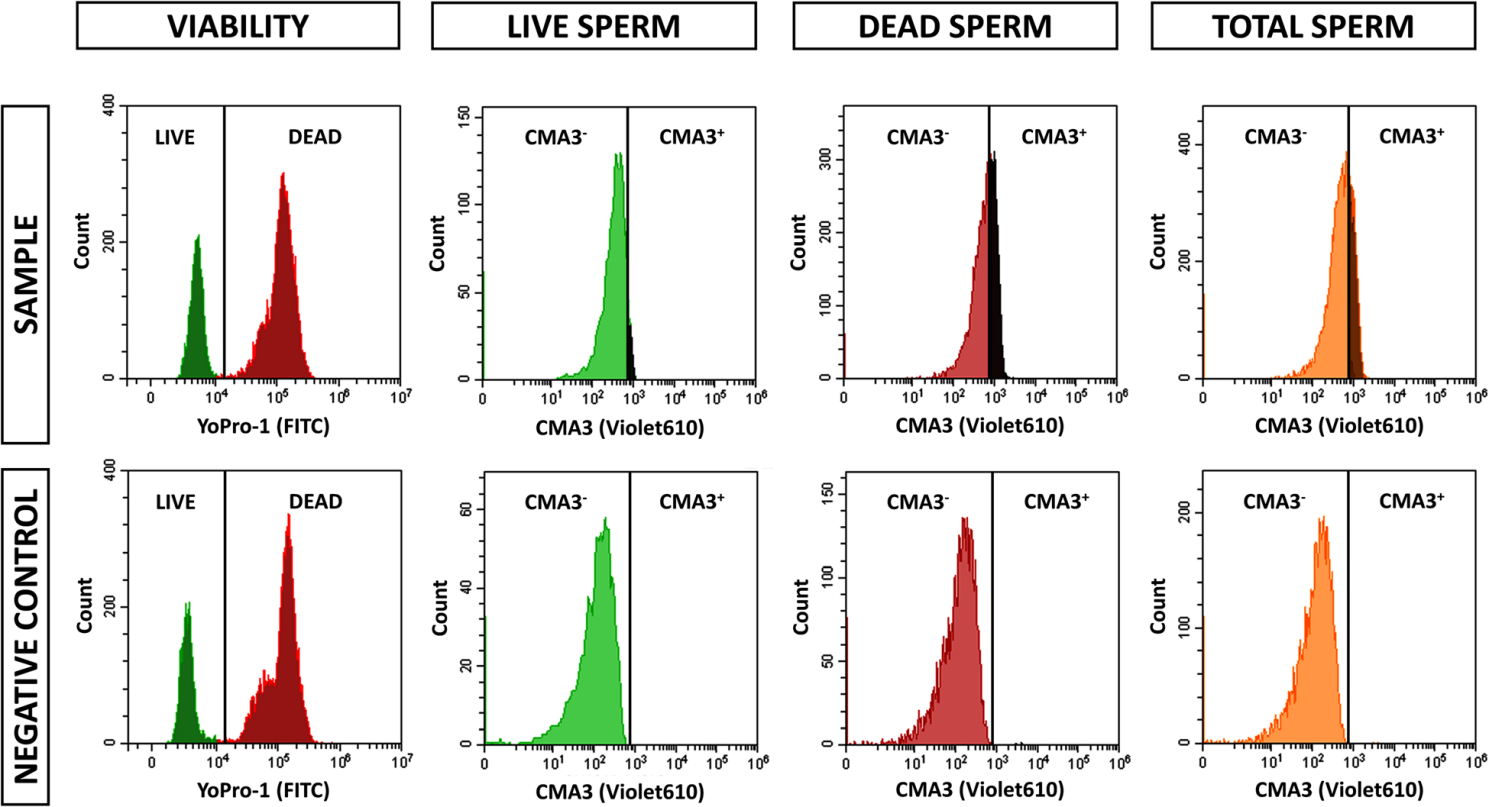
Flow cytometry histograms of a representative sperm sample and negative control without chromomycin A3 (CMA3). Sperm samples were stained with both CMA3/Yo-Pro-1, whereas CMA3 was omitted in negative controls (Yo-Pro-1 staining). Yo-Pro-1 staining was used to stablish viable (LIVE), non-viable (DEAD) and total (TOTAL; viable + non-viable sperm) populations, whereas negative control was used to stablish CMA3-positive (CMA3^+^) and CMA3-negative (CMA3^−^) populations. CMA3 histograms of the sample and negative control are represented for viable, non-viable and total sperm populations. CMA3 was acquired with the Violet610 channel (610/20), whereas Yo-Pro-1 was collected with the FITC channel (525/40)

The assessment of chromatin condensation using CMA3/Yo-Pro-1 double staining was performed by flow cytometry (Fig. 1). Yo-Pro-1, detected through FITC channel, was used to gate viable, non-viable and total sperm. Yo-Pro-1-gated populations were used to measure the percentage and fluorescence intensity of CMA3^+^ cells within each population, detected through Violet610 channel. A negative control group without CMA3 was used to establish the threshold for CMA3^+^ sperm in every sample.

Setting up of the method through incubations with 5 mmol/L DTT led to an increase in the percentage of CMA3^+^ sperm. Fluorescence intensity histograms for this experiment are depicted in Additional file 2: Supplementary Fig. 2A and results are shown in Additional file 2: Supplementary Fig. 2B. The analysis of paired samples through the Wilcoxon test revealed a statistically significant increase between untreated and treated samples (*P* < 0.05).

### Comparison of chromatin condensation between sperm populations and throughout incubation time

**Fig. 2:**
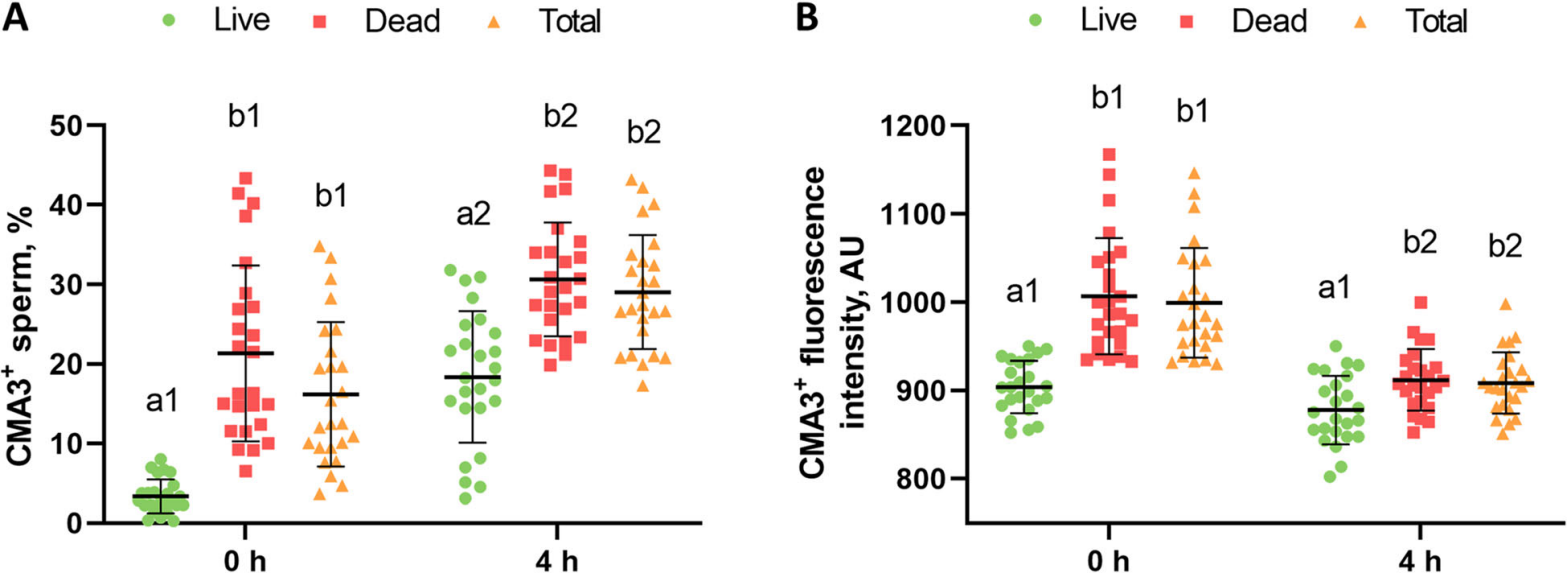
Mean ± standard deviation (SD) of the **A** percentage of chromomycin A3 (CMA3)-positive sperm (CMA3^+^ sperm) and **B** CMA3 fluorescence intensity of the CMA3-positive sperm population (CMA3^+^ fluorescence intensity [arbitrary units; AU]) of viable, non-viable and total sperm populations at 0 h and 4 h post-thaw. Samples that have different letters (a, b) indicate that statistically significant differences (*P* < 0.05) were found between sperm populations (viable, non-viable and total sperm) within a given time point (0 h and 4 h post-thaw), whereas different numbers (1, 2) indicate statistically significant differences (*P* < 0.05) between time points (0 h and 4 h post-thaw) within a given sperm population (viable, non-viable and total sperm)

The percentage of CMA3^+^ sperm in viable, non-viable and total populations after 0 and 4 h of incubation (post-thaw) is represented in Fig. 2A. The percentage (mean ± standard deviation) of CMA3^+^ sperm, after both 0 h and 4 h of thawing, was significantly lower in viable sperm (3.35% ± 2.12% and 18.37% ± 8.28%, respectively) than in both non-viable (21.33% ± 11.08% and 30.65% ± 7.17%, respectively) and total (16.19% ± 9.07% and 29.03% ± 7.18%, respectively) sperm populations (*P* < 0.05). However, no significant differences in the percentage of CMA3^+^ cells were found between non-viable and total sperm. In addition, the percentage of CMA3^+^ sperm at 0 h was significantly lower than after 4 h of thawing (*P* < 0.05) in every sperm population (viable, non-viable and total sperm). On the other hand, the CMA3 fluorescence intensity of CMA3^+^ sperm in each population is represented in Fig. 2B. Although the CMA3^+^ fluorescence intensity of viable sperm was lower than that of non-viable and total sperm at both 0 h and 4 h post-thaw (*P* < 0.05), no significant differences between non-viable and total sperm were observed (*P* > 0.05). Furthermore, the CMA3 fluorescence intensity of CMA3^+^ in non-viable and total sperm at 0 h was significantly higher than after 4 h of thawing (*P* < 0.05). However, no differences in the CMA3 fluorescence intensity of viable CMA3^+^ sperm were detected between 0 h and 4 h of incubation (*P* > 0.05).

### Correlation of sperm quality and chromatin condensation parameters with *in vivo* fertility

**Fig. 3:**
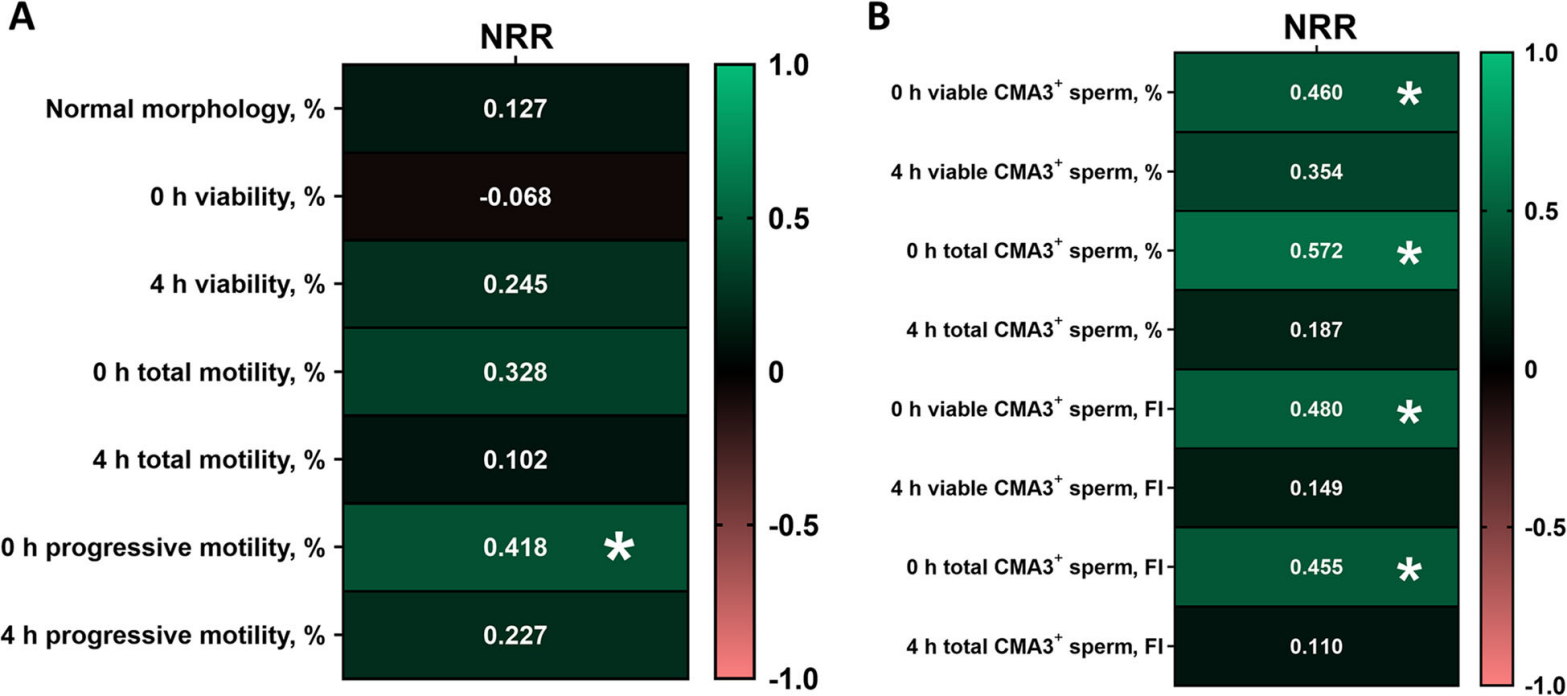
Heat map of Pearson correlation coefficients (R) between non-return rates of bulls (NRR) and **A** conventional sperm quality parameters (sperm morphology, viability, total and progressive motility) and **B** sperm chromatin condensation evaluated by chromomycin A3 (CMA3)/Yo-Pro-1 and flow cytometry, both assessed at 0 h and 4 h post-thaw. *n* = 25. * *P* < 0.05

Figure 3A and B show Pearson correlation coefficients of NRR with sperm quality parameters and chromatin condensation status, respectively, after 0 h and 4 h of thawing. Percentages of normal sperm morphology, viability and total motility did not show significant correlations with NRR either at 0 h or at 4 h post-thaw. However, the percentage of progressively motile sperm at 0 h post-thaw was significantly and positively correlated with NRR (*P* < 0.05). Regarding the relationship of chromatin condensation with *in vivo* fertility, the percentage and CMA3 fluorescence intensity of CMA3^+^ sperm in viable and total sperm populations at 0 h post-thaw showed significant and positive correlations with NRR (*P* < 0.05). Nonetheless, the same parameters after 4 h of thawing showed no correlation with NRR (*P* > 0.05).

### Comparison of sperm quality and chromatin condensation between *in vivo* fertility groups

Subfertile and high fertility groups were established using the NRR median value (Additional file 1: Supplementary Fig. 1), and conventional sperm quality parameters and chromatin condensation variables showing a significant correlation with NRR were compared between the two groups (Table 1). Although no statistically significant differences in conventional sperm quality parameters were found between subfertile and high fertility sires (*P* > 0.05), significant differences were found in their chromatin condensation status. Specifically, the percentage of viable CMA3^+^ sperm (2.46% ± 1.50% vs. 4.32% ± 2.33%; *P* < 0.05), the percentage of total CMA3+ sperm (12.06% ± 4.73% vs. 20.66% ± 10.63%; *P* < 0.05) and the CMA3 fluorescence intensity of the total CMA3^+^ sperm population (971.20 ± 35.62 vs. 1029.43 ± 71.85; *P* < 0.05) were found to differ between subfertile and high fertility bulls.

**Table 1:** Mean and standard deviations (SD) for each studied parameter and for subfertile (*n* = 13) and fertile (*n* = 12) groups

Sperm parameter at 0 h after thawing	Subfertility group	High fertility group	***P***-value
Total Fertility, % non-return to oestrus rate after 90 days	37.84 ± 1.43	40.85 ± 1.20	< 0.001 *
Normal morphology, %	89.20 ± 3.31	89.36 ± 3.07	0.900
Viability, %	50.02 ± 9.42	54.07 ± 10.79	0.327
Total motility, %	42.25 ± 15.37	51.24 ± 13.32	0.133
Progressive motility, %	24.20 ± 8.81	31.37 ± 8.66	0.052
Viable CMA3^+^ sperm, %	2.46 ± 1.50	4.32 ± 2.33	0.025 *
Total CMA3^+^ sperm, %	12.06 ± 4.73	20.66 ± 10.63	0.021 *
Viable CMA3^+^ sperm, FI	896.16 ± 29.14	915.08 ± 28.35	0.114
Total CMA3^+^ sperm, FI	971.20 ± 35.62	1,029.43 ± 71.85	0.022 *

^*^Statistical differences between groups (*P* < 0.05)

### ROC curve analysis of sperm quality and chromatin condensation parameters

**Fig. 4:**
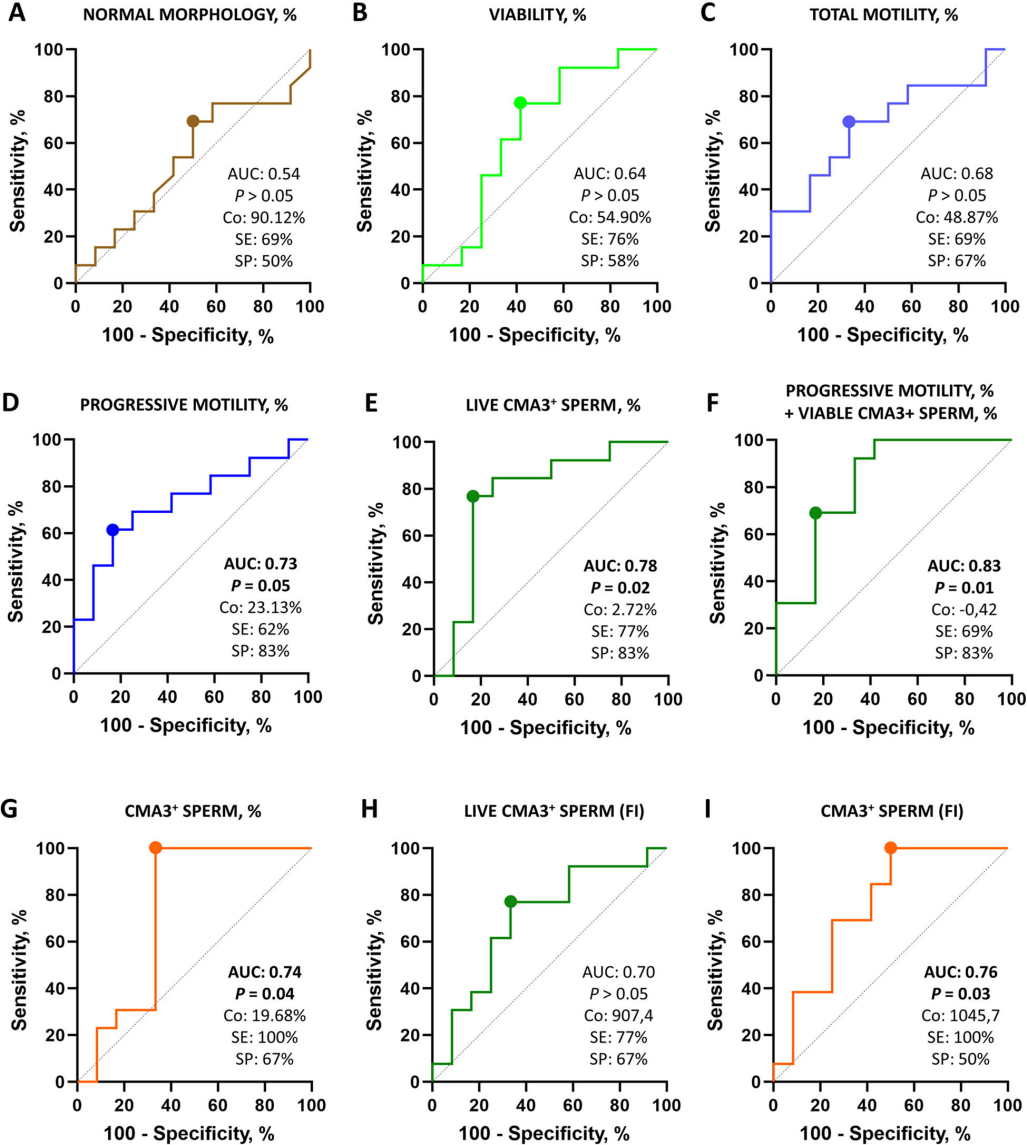
Receiver operating characteristic (ROC) curves for **A** the percentage of morphologically normal sperm; **B** the percentage of viable sperm; **C** the percentage of total motile sperm; **D** the percentage of progressively motile sperm; **E** the percentage of viable sperm (Yo-Pro-1^−^) showing chromatin decondensation (chromomycin A3-positive; CMA3^+^); **F** the percentage of progressively motile sperm and the percentage of viable sperm (Yo-Pro-1^−^) showing chromatin decondensation (CMA3^+^); **G** the percentage of total sperm showing chromatin decondensation (CMA3^+^); **H** the CMA3 fluorescence intensity of viable, chromatin-decondensed sperm (CMA3^+^/Yo-Pro-1^−^); and **I** the CMA3 fluorescence intensity of total chromatin-decondensed sperm (CMA3^+^) for discriminating between subfertile (*n* = 13) and fertile (*n* = 12) bulls. The ROC curve of each parameter shows the area under the curve (AUC) and the corresponding significance level (*p*-value; *P*), and the cut-off point (Co) of the sensitivity (SE) and specificity (SP) values

A ROC analysis was conducted using conventional sperm quality parameters and chromatin condensation variables showing a significant correlation with NRR. Thus, the capacity of morphology, viability and motility, as well as that of the percentage and fluorescence intensity of CMA3^+^ sperm in viable and total populations at 0 h post-thaw to predict *in vivo* fertility was estimated through ROC analysis (Fig. 4 and Table 2). Percentages of morphologically normal sperm, viable sperm, and total motile sperm did not show any predictive value for discriminating between fertile and subfertile individuals (*P* > 0.05). Regarding the percentage of sperm showing progressive motility, the ROC analysis set a cut-off of 23.13% to discriminate between high fertile and subfertile bulls, with a sensitivity and specificity of 61.54% (CI95% 35.52% to 82.29%) and 83.33% (CI95% 55.20% to 97.04%), respectively, and an AUC of 0.73 (CI95% 0.53 to 0.93).

**Table 2:** Receiver operating characteristic (ROC) curve analysis showing the area under the curve (AUC), standard deviation (SD), level of significance (*P*-value), cut-off value, sensitivity, specificity, positive predictive value (PPV), negative predictive value (NPV) and ODDs ratio of all sperm parameters at 0 h post-thaw for discriminating between subfertile (*n* = 13) and highly fertile (*n* = 12) bulls

Sperm parameter at 0 h after thawing	AUC	SD	***P***-value	Cut-off	Sensitivity	Specificity	PPV	NPV	ODDs ratio
Normal morphology, %	0.54 (0.30–0.77)	0.12	0.765	90.12%	69.23% (42.37–87.32)	50.00% (25.38–74.62)	60.00%	60.00%	1.39
Viability, %	0.64 (0.41–0.87)	0.12	0.231	54.90%	76.92% (49.74–91.82)	58.33% (31.95–80.67)	66.66%	70.00%	1.85
Total motility, %	0.68 (0.47–0.89)	0.11	0.128	49.87%	69.23% (42.37–87.32)	66.67% (39.06–86.19)	69.23%	66.67%	2.08
Progressive motility, %	0.73 (0.53–0.93)	0.10	0.050*	23.13%	61.54% (35.52–82.29)	83.33% (55.20–97.04)	80.00%	66.67%	3.69
Viable CMA3^+^ sperm, %	0.78 (0.57–0.98)	0.10	0.019*	2.72%	76.92% (49.74–91.82)	83.33% (55.20–97.04)	83.33%	76.92%	4.62
Viable CMA3^+^ sperm, FI	0.70 (0.49–0.91)	0.11	0.092	907.40	76.92% (49.74–91.82)	66.67% (39.06–86.19)	71.43%	72.73%	2.31
Total CMA3^+^ sperm, %	0.74 (0.51–0.96)	0.11	0.044*	19.68%	100.00% (77.19–100.00)	66.67% (39.06–86.19)	76.47%	100.00%	3.00
Total CMA3^+^ sperm, FI	0.76 (0.56–0.96)	0.10	0.030*	1045.70	100% (77.19–100.00)	50.00% (25.38–74.62)	68.42%	100.00%	2.00
Viable CMA3^+^ sperm, % + Progressive motility, %	0.83 (0.66–1.00)	0.09	0.005*	−0.42	69.23% (42.37–87.32)	83.33% (55.20–97.04)	81.82%	71.43%	4.15

With regard to the ability of chromatin condensation to predict male fertility in cattle, ROC analysis showed a significant predictive value when measured in viable and total sperm populations (*P* < 0.05). However, the AUC, sensitivity and specificity differed between sperm populations. The percentage of viable, CMA3^+^-sperm showed a sensitivity and specificity of 76.92% (CI95% 49.74% to 91.82%) and 83.33% (CI95% 55.20% to 97.04%), respectively, and an AUC of 0.78 (CI95% 0.57 to 0.98), setting the threshold at 2.72%. On the other hand, as far as the percentage of total CMA3^+^-sperm is concerned, the ROC analysis set a cut-off of 19.68%, with a sensitivity and specificity of 100% (CI95% 77.19% to 100%) and 66.67% (CI95% 39.06% to 86.19%), respectively, and an AUC of 0.74 (CI95% 0.51 to 0.96). The fluorescence intensity of CMA3^+^ sperm was also tested through ROC analysis. Although the CMA3 fluorescence intensity of viable sperm showed no predictive value for distinguishing high fertile from subfertile sires (*P* > 0.05), its value in total sperm populations allowed discriminating these two groups of bulls (*P* < 0.05), with an AUC of 0.76 (CI95% 0.56 to 0.96), a sensitivity of 100% (CI95% 77.19% to 100%) and a specificity of 50% (CI95% 25.38% to 74.62%).

Finally, a PCA of sperm parameters showing good predictive value (percentage of progressively motile and viable CMA3^+^ sperm at 0 h post-thaw) was conducted to assess their additive predictive power. The predicting value of the combined parameters for *in vivo* fertility showed the highest AUC value (0.83; CI95% 0.66 to 1.00), and a sensitivity and specificity of 69.23% (CI95% 42.37% to 87.32%) and 83.33% (CI95% 55.20% to 97.04%), respectively.

## Discussion

Recent studies evidenced the importance of paternal genetic cargo as an essential component for successful fertilization and embryo development, in both humans and domestic animals [6, 7, 9]. While sperm DNA quality has been extensively studied using DNA integrity assessments, less attention to chromatin condensation status has been paid. The majority of studies evaluating sperm chromatin condensation through CMA3 in mammalian species has been performed using fluorescence microscopy rather than flow cytometry, despite their well-known advantages [28]. Moreover, to the best of our knowledge, no studies have simultaneously evaluated CMA3 and sperm viability. Therefore, the present work established, for the first time, a protocol to evaluate sperm chromatin condensation using a simultaneous double staining (CMA3 and Yo-Pro-1) through flow cytometry. Moreover, using this technique, we evaluated sperm chromatin protamination of viable, non-viable and total sperm populations and explored the status of chromatin condensation as a new tool for predicting *in vivo* fertility in bulls.

The present study successfully applied a flow cytometry protocol that allowed assessing sperm viability and chromatin condensation simultaneously. Previous studies evaluated CMA3 in sperm by flow cytometry, using an excitation wavelength of 488 nm and fluorescence channels of 530/30 [31], 568/42 [32] and 585/42 [33–35]. However, as deoxyribonucleic acid-bound CMA3 is known to have an excitation peak of 430 nm (350 to 490 nm) and an emission peak of 590 nm (450 to 700 nm) [21], we used a 405-nm laser for excitation and collected emitting fluorescence through the Violet610 (610/20) channel. Independently, Yo-Pro-1 was excited with a 488-nm laser and acquired with the FITC (525/40) channel. Using these independent acquisition settings for both fluorochromes, we could simultaneously evaluate chromatin condensation (CMA3) and sperm viability (Yo-Pro-1) through flow cytometry.

The percentage of chromatin-decondensed sperm after 4 h of incubation was significantly higher when compared to 0 h post-thaw in every sperm population, suggesting that depromatination in frozen-thawed sperm increases over time. CMA3 is an indicator of the actual protamine content, as it competes with protamines in their binding to the minor groove of the DNA. Therefore, it can be assessed as a marker for the proper replacement of histones by protamines during spermiogenesis [36]. However, little information is present in the literature regarding the effects of cryopreservation or capacitation on sperm chromatin condensation. In this sense, a recent study reported that chromatin deprotamination occurs during sperm cryopreservation in bucks [37], probably due to capacitation-like changes. Moreover, another study reported that the sperm chromatin of bulls showing high NRR was able to better withstand the decondensation induced by EDTA and SDS [38]. Thus, it would be reasonable to suggest that the increase in chromatin decondensation after 4 h of thawing is caused by cryoinjuries or capacitation-like changes. Nevertheless, to the best of our knowledge, no specific studies have been carried out seeking for changes in chromatin condensation status during sperm capacitation.

Interestingly, although the percentage of CMA3^+^ sperm increased from 0 h to 4 h after thawing in viable, non-viable and total populations, the CMA3 fluorescence intensity only decreased in non-viable and total sperm populations. This apparent contradiction could be explained by the fact that non-viable sperm show higher levels of free radicals that preferentially oxidize 2-deoxyguanosine in the DNA leading to DNA breaks [39], as well as by the two-step hypothesis from Aitken and de Iuliis, which states that high chromatin decondensation could pave the way for free radicals to produce DNA strand breaks [40]. As CMA3-Mg^2+^ specifically sticks to guanine nucleotides, preferentially to G residues of GC-rich regions [22, 23], we posit that the presence of higher amount DNA breaks at these regions after 4 h of thawing could hinder CMA3 to bind DNA.

The double fluorochrome staining (CMA3/Yo-Pro-1) revealed significant differences in chromatin condensation between viable and non-viable sperm. At both 0 h and 4 h post-thaw, the percentage and CMA3 fluorescence intensity of non-viable sperm showing chromatin decondensation were found to be higher than those of viable sperm. Accordingly, our results evidenced lower chromatin condensation in viable than in non-viable sperm. However, while no study in the literature has simultaneously evaluated sperm viability and chromatin condensation, one could reasonably surmise that chromatin decondensation is higher in non-viable sperm due to their increased oxidative stress, as free radicals and DNA fragmentation were previously found to be positively correlated with sperm chromatin decondensation [15, 41].

From all conventional sperm quality parameters tested (sperm morphology, viability, total and progressive motility), only progressive motility at 0 h post-thaw did show a significant and positive correlation with *in vivo* fertility, thus appearing to be a useful tool for differentiating between highly fertile and subfertile individuals. However, when comparing percentages of progressively motile sperm between subfertility and high fertility groups, no statistically significant differences were found. Motility is the most widely used measure for estimating sperm quality and, in fact, other studies previously reported positive correlations between motility and bull fertility using both subjective and objective assessments [42, 43]. On the other hand, the percentage and CMA3 fluorescence intensity of viable and total sperm populations at 0 h, but not at 4 h post-thaw, were found to be positively correlated with *in vivo* fertility. Accordingly, the percentages of CMA3^+^ sperm in viable and total sperm populations, as well as the CMA3 fluorescence intensity of total sperm at 0 h post-thaw were found to be higher in highly fertile individuals when compared to subfertile males. Moreover, considering that previous studies performed in human sperm reported negative correlations [25, 44], it is worth noting that all parameters evaluating chromatin decondensation herein were positively correlated with *in vivo* fertility in bulls. Although little information in the literature regarding the relationship between chromatin condensation and male fertility in livestock is found, a previous study evaluating CMA3 by fluorescence microscopy in cryopreserved sperm also described higher levels protamine deficiency in bulls showing high *in vitro* fertility rates [26]. Additionally, another study evaluating the relationship between chromatin condensation (CMA3) evaluated by fluorescence microscopy and *in vivo* fertility (NRR) in bulls did not report significant correlations [38]. It is important to highlight that, while CMA3 studies in humans were carried out using fertile and infertile men, those in farm animals utilised fertile and subfertile males, since fertility-based selection virtually eradicates male infertility in these species. For this reason, we speculate that infertile individuals could have depicted higher incidence of chromatin decondensation. In this regard, it is worth mentioning that no research involving human fertile donors from semen banks to correlate CMA3^+^ sperm with fertility rates has been conducted.

In the present study, ROC analyses were performed to compare the predictive value of conventional sperm quality parameters and chromatin condensation status determined with CMA3/Yo-Pro-1 staining and flow cytometry for discriminating subfertile and highly fertile bulls. Thus, the AUC, sensitivity, and specificity of conventional sperm quality parameters and CMA3/Yo-Pro-1 assay were subsequently calculated. Regarding conventional sperm quality parameters, whereas the percentage of morphologically normal sperm, the percentage of viable sperm and the percentage of total motile sperm showed no significant predictive value for male *in vivo* fertility, the percentage of progressively motile sperm did. At a cut-off point of 23.13%, the test yielded the highest sensitivity and specificity of 61.54% and 83.33%, respectively. Thus, classifying potential fertility of bulls based on their progressive motility would result in high rates of false negatives. In contrast, chromatin condensation assessed by CMA3/Yo-Pro-1 staining showed better predictive value than conventional sperm quality parameters. The percentage and CMA3 fluorescence intensity of total CMA3^+^ sperm showed a significant predictive value for *in vivo* bull fertility. Nevertheless, although the rate of false negatives is very low for these parameters, the rate of false positives was seen to be very high (33% to 50%). However, when the percentage of chromatin-decondensed sperm was measured within the viable sperm population, its predictive value for *in vivo* bull fertility was higher. At a cut-off point of 2.72%, the test yielded the highest sensitivity and specificity of 76.92% and 83.33%, respectively, thus exhibiting 23.08% of false negative and 16.67% of false positive rates. Interestingly, the additive predictive value of the percentage of progressively motile and viable CMA3^+^ sperm showed a higher AUC but lower sensitivity and identical specificity than viable CMA3^+^ sperm. For this reason, the percentage of viable sperm showing chromatin decondensation (CMA3^+^/Yo-Pro-1^−^ staining) should be considered as a biomarker for *in vivo* fertility in bulls, since it presents higher accuracy and precision than the overall proportions of CMA3^+^ sperm or conventional sperm quality parameters such as progressive motility. However, although promising, these preliminary results require further validation using larger a sample size in order to confirm CMA3/Yo-Pro-1 as a reliable *in vivo* fertility biomarker.

## Conclusions

The present study established a protocol to determine sperm chromatin condensation status through a simultaneous double staining (CMA3 and Yo-Pro-1) using flow cytometry. This novel approach to the evaluation of sperm chromatin condensation status evidenced, for the first time, significant differences in chromatin protamination between viable and non-viable sperm, being significantly lower in the latter than in the former. Finally, sperm chromatin condensation of viable sperm assessed by CMA3/Yo-Pro-1 staining and flow cytometry was described here as a new tool for predicting *in vivo* fertility in bulls, showing better accuracy and precision than conventional sperm quality parameters. This suggests that the use of this biomarker could maximise the efficiency of the dairy breeding industry.

## Supplementary Information

**Additional file 1 Supplementary Fig. 1**. Distribution of 90-days non-return rates (NRR) of the bulls used in the study (*n* = 25). Red bars: subfertile group (NRR < 39.4). Green bars: high fertility group (NRR > 39.4).

**Additional file 2 Supplementary Fig. 2**. Set up of chromomycin A3 (CMA3) labelling in bull untreated samples and samples treated with 5 mmol/L Dithiothreitol (DTT) for 45 min. Negative controls without CMA3 for each sample were included in order to set up the threshold value for positive cells. Orange parts indicate positive CMA3 cells. (**A**) Flow cytometry histograms for fluorescence intensity (FI) at 610 nm; (**B**) Data from the five bull sperm samples used to set up the experiment.

## Abbreviations

AI: Artificial insemination
AUC: Area under the curve
CI95: 95% of the confidence interval
CMA3: Chromomycin A3
NPV: Negative predictive value
NRR: Non-return rate
PPV: Positive predictive value
ROC: Receiver operating characteristic curve
SD: Standard deviation
