# Supplementary figures and images for: Sperm chromatin condensation as an in vivo fertility biomarker in bulls: a flow cytometry approach
Source: J Anim Sci Biotechnol. 2021 Nov 9;12:115. doi: 10.1186/s40104-021-00634-7 (PMC8576882; doi:10.1186/s40104-021-00634-7)

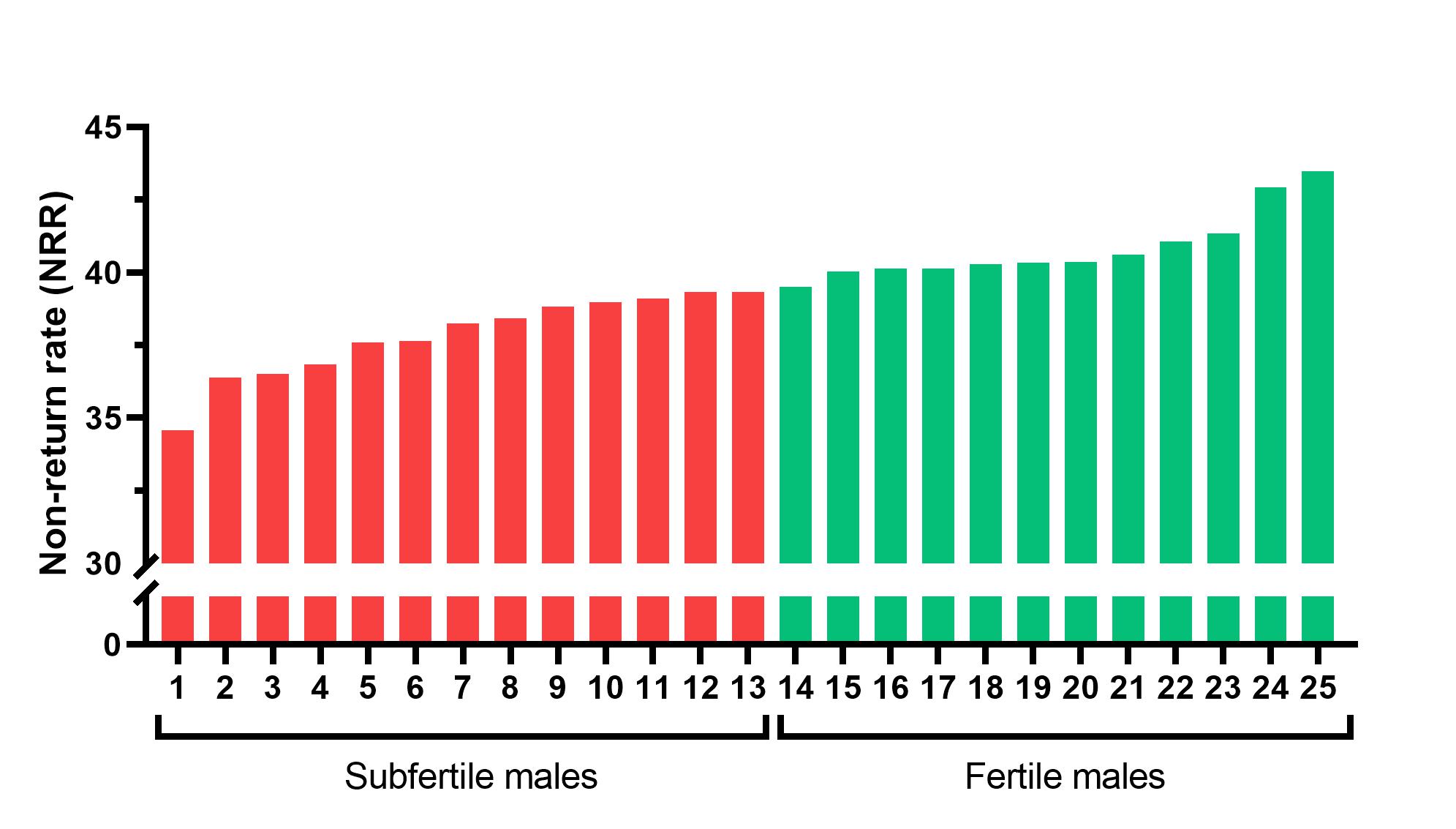

Supplement: Supplementary file 1 — Additional file 1 Supplementary Fig. 1. Distribution of 90-days non-return rates (NRR) of the bulls used in the study (n = 25). Red bars: subfertile group (NRR < 39.4). Green bars: high fertility group (NRR > 39.4). [file 40104_2021_634_MOESM1_ESM.jpg]

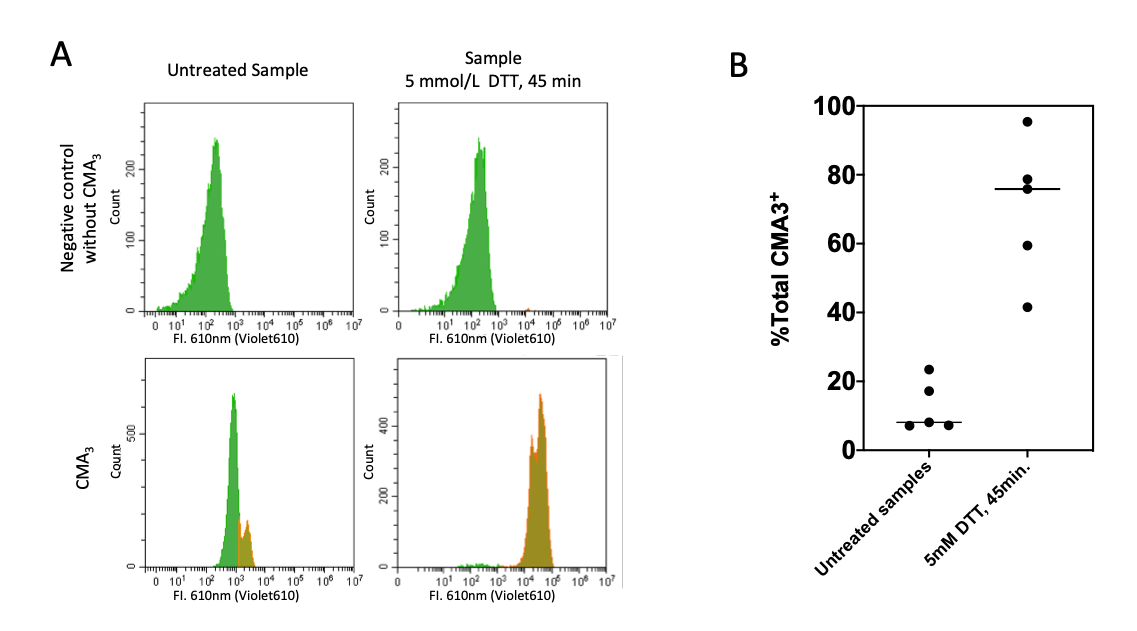

Supplement: Supplementary file 2 — Additional file 2 Supplementary Fig. 2. Set up of chromomycin A3 (CMA3) labelling in bull untreated samples and samples treated with 5 mmol/L Dithiothreitol (DTT) for 45 min. Negative controls without CMA3 for each sample were included in order to set up the threshold value for positive cells. Orange parts indicate positive CMA3 cells. (A) Flow cytometry histograms for fluorescence intensity (FI) at 610 nm; (B) Data from the five bull sperm samples used to set up the experiment. [file 40104_2021_634_MOESM2_ESM.tif]
